# Fluorination of Diamond Nanoparticles in Slow Neutron Reflectors Does Not Destroy Their Crystalline Cores and Clustering While Decreasing Neutron Losses

**DOI:** 10.3390/ma13153337

**Published:** 2020-07-27

**Authors:** Alexei Bosak, Artur Dideikin, Marc Dubois, Oleksandr Ivankov, Egor Lychagin, Alexei Muzychka, Grigory Nekhaev, Valery Nesvizhevsky, Alexander Nezvanov, Ralf Schweins, Alexander Strelkov, Alexander Vul’, Kirill Zhernenkov

**Affiliations:** 1 European Synchrotron Radiation Facility, 71 Avenue des Martyrs, F-38042 Grenoble, France; alexei.bossak@esrf.fr; 2 Ioffe Institute, Ru-194021 St. Petersburg, Russia; Dideikin@mail.ioffe.ru (A.D.); AlexanderVul@mail.ioffe.ru (A.V.); 3 SIGMA Clermont (ICCF), Université Clermont Auvergne, 24 Avenue Blaise Pascal, F-63178 Aubière, France; marc.dubois@uca.fr; 4 Joint Institute for Nuclear Research, 6 Joliot Curie, Ru-141980 Dubna, Russia; ivankov@jinr.ru (O.I.); lychag@nf.jinr.ru (E.L.); muz@nf.jinr.ru (A.M.); grigorijnekhaev@yandex.ru (G.N.); alexnezv@gmail.com (A.N.); str@jinr.ru (A.S.); k.zhernenkov@fz-juelich.de (K.Z.); 5 Research Center for Molecular Mechanisms of Aging and Age-Related Diseases, Moscow Institute of Physics and Technology, 9 Institutskiy per., Ru-141701 Dolgoprudny, Russia; 6 Institute for Safety Problems of Nuclear Power Plants NAS of Ukraine, 12 Lysogirska str., Ukr-03028 Kyiv, Ukraine; 7 Faculty of Physics, Lomonosov Moscow State University, Leninskie Gory, Ru-119991 Moscow, Russia; 8 Department of Nuclear Physics, Dubna State University, Universitetskaya 19, Ru-141982 Dubna, Russia; 9 Institut Max von Laue-Paul Langevin, 71 av. des Martyrs, F-38042 Grenoble, France; schweins@ill.eu; 10 Jülich Centre for Neutron Science at Heinz Maier-Leibnitz Zentrum, Forschungszentrum Jülich GmbH, 85748 Garching, Germany

**Keywords:** powder of diamond nanoparticles, neutron reflector, fluorination

## Abstract

If the wavelength of radiation and the size of inhomogeneities in the medium are approximately equal, the radiation might be intensively scattered in the medium and reflected from its surface. Such efficient nanomaterial reflectors are of great scientific and technological interest. In previous works, we demonstrated a significant improvement in the efficiency of reflection of slow neutrons from a powder of diamond nanoparticles by replacing hydrogen located on the surface of nanoparticles with fluorine and removing the residual *sp*^2^ amorphous shells of nanoparticles via the fluorination process. In this paper, we study the mechanism of this improvement using a set of complementary experimental techniques. To analyze the data on a small-angle scattering of neutrons and X-rays in powders of diamond nanoparticles, we have developed a model of discrete-size diamond nanospheres. Our results show that fluorination does not destroy either the crystalline cores of nanoparticles or their clustering in the scale range of 0.6–200 nm. This observation implies that it does not significantly affect the neutron scattering properties of the powder. We conclude that the overall increase in reflectivity from the fluorinated nanodiamond powder is primarily due to the large reduction of neutron losses in the powder caused by the removal of hydrogen contaminations.

## 1. Introduction

Neutron scattering is an indispensable tool for both fundamental and applied research. As such there is a pronounced worldwide effort to increase the range of useful neutrons towards smaller velocities (larger wavelengths), driven in particular by largescale structure diffractometers, reflectometers, time-of-flight and spin-echo techniques, fundamental particle physics, etc. However, the progress in the field is limited by the severe flux decrease of available slow neutrons. The properties of neutron reflectors cause this dramatic decrease of slow neutron flux from present sources, nuclear reactors, and spallation sources. Independently of the choice of materials, their evident common feature is that they are composed of atoms separated by distances of ~10^−1^ nm. As soon as the neutron wavelength reaches this value, neutrons penetrate through the reflector and are lost. For example, filters made of pyrolytic graphite pass neutrons with the wavelength of 2.4 Å but intensively scatter neutrons with the wavelength of 1.2 Å. The present work is a part of a broader scientific program, and we pursue to overcome this limitation by developing a novel type of neutron reflectors. It is based on the coherent enhancement of elastic scattering of slow neutrons in nanostructured media.

We study a particular case of wave scattering in inhomogeneous media [1], and namely neutron diffusion [2,3,4,5]. In general terms, due to the multiple scattering on inhomogeneities, radiation diffuses in such a medium and can reflect from its surface. For different types of radiation, examples are the reflection of electromagnetic waves from inhomogeneities of the atmosphere, aerosols, rain, snow, biological issues, and composite materials [6]. Charged particles (protons, electrons) or neutral particles (atoms) provide other numerous examples of such wave reflection.

Following this analogy, we found that powders of detonation nanodiamonds (DNDs) [7,8] with typical nanoparticle sizes of 4–5 nm reflect very cold neutrons (VCNs) diffusively at any incidence angle [9,10,11,12], and they quasi-specularly reflect cold neutrons (CNs) at small incident angles [13,14]. In both cases, DNDs have provided a record reflectivity compared to other known media. This is due to the exceptional combination of the large coherent scattering length of carbon (C) (b^C^_c.sc._ = 6.65 fm, the corresponding coherent scattering cross section is σ^C^_c.sc._ = 5.55 b), the high volume density of diamond (ρ^C^ = 3.5 g/cm^3^), the low neutron losses (the absorption cross section of σ^C^_abs_ = 3.5 mb, and low inelastic scattering cross sections depending on the temperature). Nanodiamonds of similar size and properties, but with a smaller amount of impurities, can also be produced through laser synthesis [15]. The geometric sizes and shapes [16] of DNDs are important for optimizing the specific properties of such reflectors, which in turn depend on applications [17,18]. Scattering cross sections have been studied in detail in references [19,20,21], which in particular underlined the importance of clustering/agglomeration of DND powder samples to allow for a proper interpretation of neutron scattering experiments, and the virtual absence of inelastic scattering. Significant efforts have been devoted to including the diffusion of slow neutrons in the DND powders to neutron transport simulations [21,22,23].

Hydrogen (H) in DNDs is an important cause of neutron losses that reduces the efficiency of the reflection. H is present in raw DNDs in large quantities (on average one H atom per 7.4 ± 0.2 C atoms), and it has a large absorption cross section (σ^H^_abs_ = 0.33 b) and an exceptionally large incoherent scattering cross section (σ^H^_in.sc._ = 108 ± 2 b at room temperature) that consists of inelastic and elastic contributions depending on the temperature [24]. In DNDs, H atoms are involved in C-OH, C-H, CH_2_, and COOH groups [25,26].

Recently, we applied a chemical treatment to DNDs, namely a gas (F_2_)-solid fluorination [27,28,29] which reduced the quantity of hydrogen by the factor of ~30 (on average only one H atom remains per 430 ± 30 C atoms in freshly fluorinated DNDs) and provided a significantly higher efficiency of quasi-specular reflection of neutrons [30]. In this paper, we report results of our study of the mechanism of this improvement (using small quantities of samples <50 mg). In the future, we are going to use the knowledge gained from this study to produce much larger quantities of DNDs with designed parameters, needed for full-scale reflectors. Alternative approaches to produce DNDs with a reduced content of H are its deuteration or modifications of the production conditions of DNDs in order to avoid large hydrogen quantities. Deuterated DNDs seem to be unstable relative to the substitution of D by H. Modifications of the production procedure have been studied in reference [31].

Here, we do not reproduce the theoretical formalism used to analyze the interaction of neutrons with DNDs in powder form as it was presented in our previous publications, for instance in reference [30].

In Section 2, we describe the samples (DNDs and F-DNDs), and discuss the choice of methods used to characterize them. In Section 3, we present our experimental results. In Section 4, we compare these experimental results to our preceding studies, discuss the effects of fluorination on DND scattering properties, and prospects of this study. In Section 5, we draw our conclusions. 

## 2. Materials and Methods

### 2.1. Samples

To compare the results of this study directly with the results obtained in our previous works [27,28,29,30], we used DND powders of the same type: Raw DNDs produced at the Federal State Unitary Enterprise “Russian Federal Nuclear Center – Academician E.I. Zababakhin All-Russian Research Institute of Technical Physics” (FSUE “RFNC-VNIITF” institute), Snezhinsk, in accordance with the procedure described in the Technical Regulations TY 2-037-677-94 as an initial material, and fluorinated nanodiamonds (F-DNDs) of the same type.

**Figure 1:**
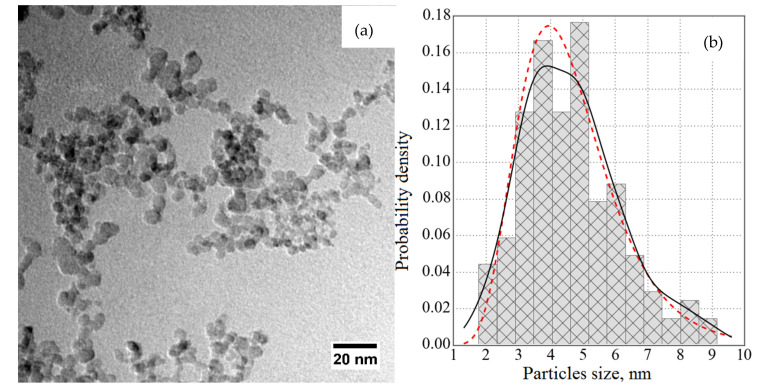
(**a**) A TEM image of the fluorinated nanodiamonds (F-DND) sample, (**b**) a corresponding diameter distribution of the DNDs. The red dashed line corresponds to the lognormal distribution. The black solid line indicates a data fitting.

Figure 1 shows an image of F-DNDs measured using a transmission electron microscope (TEM, FEI Tecnai G2 30 S-TWIN, NRC “Kurchatov Institute” – CRISM “Prometey”, St. Petersburg, Russia) and a size distribution of DNDs evaluated using these data. A 2–3 mm^3^ sample of the powder was added to 1 mL of distilled water, and the container with the mixture was placed in an ultrasonic bath filled with water. It was sonicated for 15 min. The resulting suspension (2–3 drops) was applied to a carbon replica placed on the copper grid. After drying, the replica was examined via TEM.

During fluorination of carbonaceous materials, the C hybridization changes from *sp*^2^ to *sp*^3^ through the formation of covalent C–F bonds. The higher the crystalline order for graphitic carbons, the higher the fluorination temperature is. Since the *sp*^2^ C shell on diamond cores is disordered, it is decomposed with F_2_ gas at a high temperature (>450 °C). CF_4_ and C_2_ F_6_ gases are then formed. On the contrary, diamond cores do not react with F_2_ molecules because the C atoms are stabilized both in a *sp*^2^ hybridization and in the stable (crystalline) C lattice. Moreover, F forming covalent C–F bonds may replace H atoms bonded to *sp*^3^ C in CH, CH_2_, or C–OH groups.

Below we list the known properties of DND powders that are relevant to this study.

It is a well-known fact that DND powder produced by the detonation synthesis (usually from trinitrotoluene and hexogen) contains particles that have a diamond crystal lattice and a characteristic size of 4–5 nm. The unusually narrow range of sizes of diamond nanocrystals has been emphasized by numerous researches [25,32,33,34] and explained by assuming that it is diamond, rather than graphite, that appears to be a thermodynamically stable form of nanocarbon when the particle size lies in the above mentioned range. This hypothesis was proposed in reference [35] and supported by the results of numerical simulations of a stable C cluster [36].

The structure of a DND particle is usually represented in the core-shell model [8] as a diamond core (*sp*^3^ hybridization) in the form of a polyhedron [37] surrounded by a non-crystalline C shell (with *sp*^3^-*sp*^2^ hybridization of C atoms). The shell contains C–H_x_, C–C–H, C–OH, C–O, C=C [26]. The shell thickness is 0.4–1.0 nm [38,39,40]. The fraction of H atoms chemically bound to the surface is ~5%, which corresponds to the results of references [24,27]. At the same time, it should be emphasized that DNDs supplied by different manufactures differ significantly in the structure and chemical composition of the shell that covers the crystalline diamond core, and in particle sizes.

Industrial DND powder contains strong agglomerates (agglutinates) with mean sizes ranging from 40 to 200 nm and consisting of 4–5 nm crystalline grains. Deagglomeration processes have been just recently developed firstly by mechanical milling [41,42] then by the annealing process in different atmospheres [43,44]. In dried industrial powders, individual DND particles form a hierarchical structure of agglutinates [42,45,46]. The next level of agglomerates have an average size of more than 1 μm and can be easily destroyed [47].

DNDs are highly hygroscopic. As a matter of fact, in the atmosphere of air with the humidity of ~60%, the amount of H in DNDs doubles [24]. This is mostly due to water molecules adsorbed on the surface of DNDs containing H. Hydrophilic properties of DND are presumably due to the –OH and –COOH groups residing on the surface. Hard fluorination replaces H by F thus increasing the hydrophobicity.

Our measurements showed that the hygroscopicity of the freshly produced F-DND powder is 30 times lower than that of DNDs before the fluorination. The substitution of H by F on the DND surface explains such a behavior.

Agglomeration of DNDs not only leads to an increase in the size of scattering objects but also modifies the pores between DNDs. Neutron scattering by a vacuum cavity of a certain size located in a continuous medium resembles scattering by a particle of this substance of the same size in a vacuum. Pore sizes include values both significantly smaller and larger than the sizes of individual DNDs [48].

One of the basic principles of the operation of DND neutron reflectors is the fact that coherent neutron scattering by atoms of a nanoparticle is strongly enhanced, while the inelastic scattering is not. Therefore, neutron scattering by DNDs is mainly elastic, and the dynamics of the system does not play its role. Therefore, the scattering and transfer of neutrons in DND powders is determined by properties and the relative position of DNDs, and not by the strength of the physical and chemical bonds between them. In this paper, we distinguish agglomerates and clusters. By clusters, we mean the areas of denser packing of DNDs as only such clusters are relevant to neutron scattering. In the zeroth approximation, the relevant parameter is the average volume density of the cluster. The properties of clusters that are important for the diffusion and transport of neutrons might not coincide with properties evaluated by other methods.

### 2.2. Rationale for the Choice of Experimental Methods

Many methods can be used in material and neutron science to measure the sizes of DNDs and their clusters in DND powders. They include neutron (SANS) and X-ray (SAXS) small-angle scattering, neutron (ND) and X-ray (XRD) diffraction, neutron quasi-specular reflection (NQSR), TEM, scanning electron microscopy (SEM), dynamic light scattering (DLS), etc. They provide fundamentally different information and can only be used to address specific questions.

SANS provides the most direct and unambiguous information for the analysis of scattering and transport of slow neutrons in DND powders. It is sensitive to both individual DNDs and their clusters. The results of SANS measurements are presented in Section 3.1. As the probability and angular characteristics of scattering depend on the neutron velocity, one has to use models for applying the measured results to other neutron velocities and scattering angles. In this work, we propose a model of discrete-size diamond nanospheres. It is described in Section 3.2.

**Figure 2:**
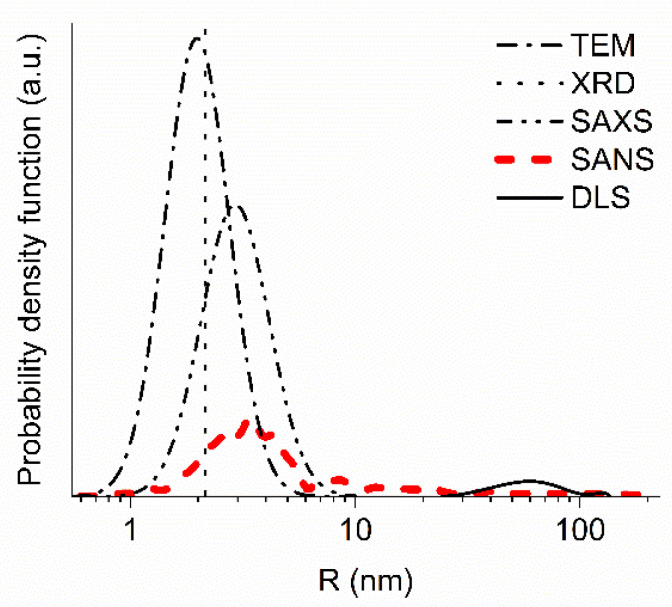
The probability density as a function of radius (nm) evaluated using neutron small-angle scattering (SANS) (red thick dashed line), X-ray small-angle scattering (SAXS) (black thin dash-double-dotted line), TEM (black thin dash-dotted line), and dynamic light scattering (DLS) (black thin solid line). The vertical black dotted line indicates a mean radius measured with XRD. All measurements are performed with F-DND samples, except for SAXS, which is performed with a DND sample. The details of the different measurements are given below in the text.

SAXS is sensitive to the electronic structure of the substance, but not to the neutron-optical potential (such as SANS is). In particular, the contribution of H atoms to SAXS is much smaller than it is to SANS, while the contribution of heavy metallic impurities is relatively higher. However, in the case of DND powders, SAXS and SANS give similar results, because the scattering is defined by C atoms, while other impurities are small. Figure 2 gives an example of the result obtained from SAXS data.

Methods of diffraction and small-angle scattering of neutrons and X-rays are complementary to each other and provide detailed information on scattering properties of powder samples.

ND and XRD provide similar information, and are sensitive to the crystal structure of diamond cores. These methods are not sensitive to DND *sp*^2^ shells and clusters. XRD results are presented in Section 3.3.

NQSR at time-of-flight reflectometers provides simultaneously the information contained in all these methods. However, multiple neutron scattering, which is important for implementing this method, requires the use of one or another model to analyze the results.

TEM and SEM serve for the visualization of the powder structure. These methods allow evaluating both the sizes of DNDs (including *sp*^2^ shells) and the sizes and structure of clusters. However, the accurate determination of size distributions is more difficult than it is in the case of neutron and X-ray scattering because it requires the analysis of a large number of images. Examples of TEM and SEM images can be found in Figure 1 and Figure 6.

DLS is less effective for studies of DND neutron reflectors, because it studies a fundamentally different phenomenon. It is sensitive to the motion of individual DNDs and their agglomerates in liquid dispersions as a whole. In addition, the agglomerates of DNDs in liquid dispersions and ones in the initial dry powders are different [19]. In addition, the size of DNDs obtained this way is overestimated as it includes water layers entrained by the DND as it moves. Even if there is only a small fraction of large particles, the mean size obtained may appear larger than the real one [49]. 

To illustrate this comparison of the methods, in Figure 2 we present the results obtained by the SANS, SAXS, XRD, TEM, and DLS methods. SANS, SAXS, XRD, and TEM give compatible results. Two or more closely spaced DNDs can produce nearly the same SANS and SAXS images as a single larger DND [6]. This effect, apparently, explains the larger sizes obtained by the SANS and SAXS methods. Note that in the model of discrete-size diamond nanospheres presented in Section 3.2 as well as in the majority of other models that describe SANS and SAXS data, such interference effects are ignored. The difference in the estimation of the mean size of scatterers for all these methods (SANS, SAXS, XRD, TEM), on the one hand, and DLS, on the other hand, is of an order of magnitude. It is related to the fact that they characterize different details of the DND structure. The value of 50–100 nm measured by DLS is typical for the size of agglomerates [44,50].

## 3. Experimental Results

### 3.1. Small-Angle Neutron Scattering

We measured SANS on powder samples using two instruments: The D11 instrument at ILL [51,52,53] and the YuMO time-of-flight spectrometer in a two-detectors mode at FLNP, JINR [54]. The neutron wavelength at D11 was 6 Å and the range of transferred momenta of 10^−2^ nm^−1^ < Q < 10^0^ nm^−1^. The neutron wavelengths at YuMO were 0.7–5.0 Å and the range of transferred momenta of 7 × 10^−2^ nm^−1^ < Q < 10^1^ nm^−1^. The measured scattering curves for the YuMO spectrometer were corrected for the background scattering from the empty cuvette. The absolute calibration of the scattered intensity was made using the vanadium standard in the SAS program [55].

The samples were placed inside standard high precision cells made of Quartz SUPRASIL with the light path of 1 mm at D11 and dur-aluminum cells with the light path and windows thickness of 1 mm at YuMO. The sample density of both types were equal to each other, 0.24 ± 0.01 g/cm^3^, at D11, and it was equal to 0.33 ± 0.01 and 0.36 ± 0.01 g/cm^3^ at YuMO for DNDs and F-DNDs, respectively. The powder compaction by tapping explains its higher density for the use at YuMO. 

**Figure 3:**
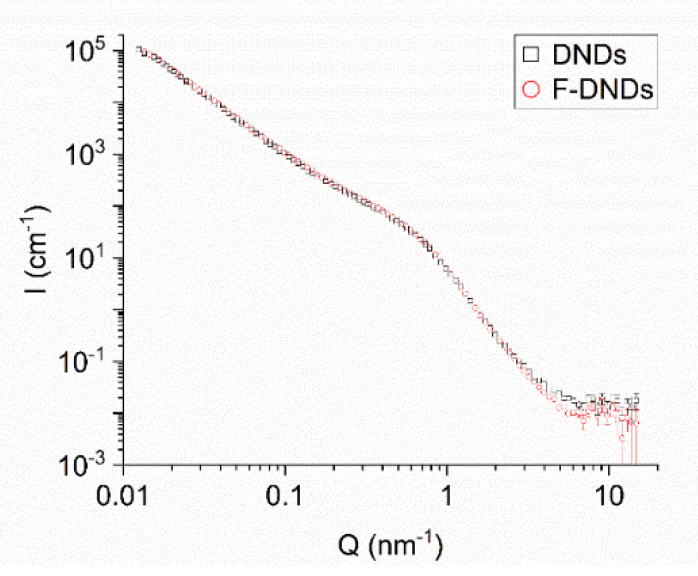
Measured intensity I (cm^−1^) of scattered neutrons as a function of the transferred momentum Q (cm^−1^) for DND and F-DND samples shown with black squares and red circles, respectively. For the convenience of comparing the results, the data are normalized to the equal sample mass.

Figure 3 shows the neutron scattering intensity as a function of the transferred momentum. The data from D11 and YuMO have been merged. In the intersecting range of transferred momenta, two sets of data coincide within the experimental accuracy. Scattering curves for two types of samples are indistinguishable in almost the entire range of transferred momenta; a minor difference is observed only for Q > 3 × 10^0^ nm^−1^. This observation means that fluorination does not significantly affect the primary clusters in the radii range of 0.6–200 nm, as well as the neutron scattering of individual DNDs. This result is important for two reasons. First, fluorination removes *sp*^2^ C, and we are interested in how it affects clustering. Second, fluorination replaces at least 8% [27] of atoms in the powder with atoms which the nuclei have significantly different scattering lengths (−3.74 and 5.64 fm, for ^1^ H and ^19^ F, respectively).

As effects of both these factors appeared to be virtually absent, we performed a triple check of the reliability of this conclusion. In addition to the described measurements at D11 and YuMO, we performed another measurement at D11 with another pair of samples of the same type. The result of this experiment confirmed the almost identical scattering curves, with a slightly higher total probability of neutron scattering for the F-DND sample. This conclusion is in apparent contradiction to the results of reference [56], which states that fluorination destroys agglomerates. In fact, these two works study agglomerates/clusters of different characteristic sizes. While we are interested in the sizes of 0.6–200 nm, which are of importance for the diffusion of slow neutrons in DND powders, the authors of reference [56] measured larger objects, which are indeed more easily destroyed.

### 3.2. A Model of Discrete-Size Diamond Nanospheres

For a quantitative analysis of neutron and X-ray scattering on DNDs and their clusters, we developed the following model of discrete-size diamond nanospheres. We simulated both the DNDs and their clusters with diamond nanospheres. We assumed a discrete set of nanosphere sizes, in which the next generation nanosphere radius is larger than the previous one by a certain factor. In the calculations presented below, the radii are uniformly distributed on a logarithmic scale, with 20 values of the radius by an order of magnitude. 

**Figure 4:**
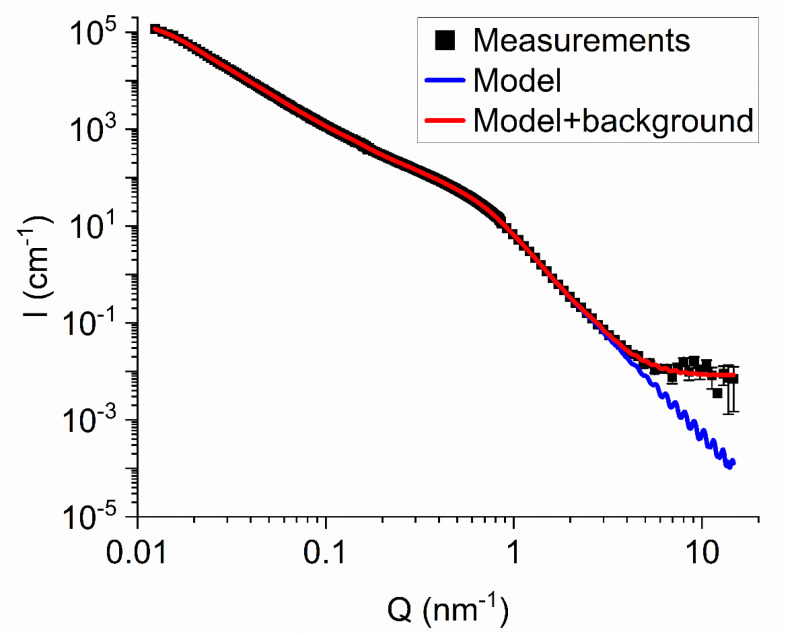
Comparison of measured and simulated intensity I (cm^−1^) of neutron scattered as a function of the transferred momentum Q (cm^−1^) for the F-DND sample. Black squares denote the experimental data. The thin blue line shows the results of the simulation within the model of discrete-size diamond nanospheres, and the thick red line contains in addition the background intensity of 9·10^−3^ nm^−1^.

We adjusted populations of DNDs/clusters of each generation to fit the experimental data. The fitting procedure is stable with respect to the parameter choice of the model (the initial populations of cluster generations, the step of increasing the mass of clusters of the next generation, the boundaries of the mass range if it sufficiently broad). The results of simulations for the two data sets, for DNDs and F-DNDs, are practically indistinguishable. Figure 4 illustrates the comparison of measured and simulated intensities of SANS from F-DND samples.

**Figure 5:**
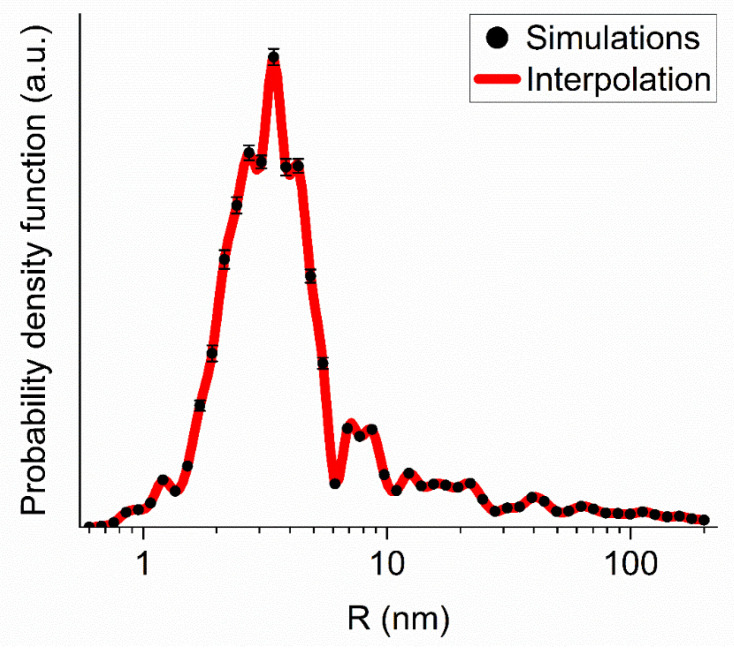
The probability density as a function of radius (in nm) evaluated within the model of discrete-size diamond nanospheres for the F-DND sample. Red circles show the simulation results. The red solid line interpolates the simulation results.

Figure 5 shows the mass distribution of populations of DNDs and clusters of different generations evaluated within the model of discrete-size diamond nanospheres for the F-DND sample. Although the mass fraction contained in large clusters is relatively small, they determine the neutron scattering in the region of small transferred momenta Q shown in Figure 3 and Figure 4.

When analyzing the size distribution of diamond nanospheres, it should be taken into account that actually existing clusters are significantly different from the diamond density and the shape that does not coincide with the ideal nanosphere. The neutron scattering cross section on a real cluster is smaller than the scattering cross section on an ideal diamond nanosphere. Therefore, the number of clusters is greater than that obtained in our model as many times as the scattering cross sections differ. On the other hand, to calculate neutron diffusion in diamond nanopowders, the diamond nanosphere model gives a good approximation. Respectively, the total mass concentrated in diamond nanospheres, estimated in the framework of our model, turns out to be lower than the total mass of the powder. This feature in its effective form represents the presence of C in non-diamond phases (*sp*^2^), non-C elements, scattering by pores, the difference between the shape of diamond cores of DNDs and ideal nanospheres, interference of scattering on neighbor DNDs, etc. In a sense, this simple model of clustering is analogous to expanding of a mathematical function in a series into simple basis functions. The amplitude of each term of the decomposition does not directly reflect the physical reality but allows analyzing the overall behavior of the investigated function. The effective mass of the powder, evaluated this way, is its useful characteristic. It yields the mass fraction of powder that is effectively involved in neutron scattering. The remaining mass is effectively involved in neutron loss, but does not participate in the scattering. The larger the effective mass, the greater the efficiency of the DND powder for neutron reflection is. For this particular F-DND sample and its conditioning used, the effective mass is ~63%; for DNDs, it is by the way nearly the same.

These results show that DND and F-DND powders are the same in the radii range of powder structures from 0.6 to 200 nm. The maximum sizes of agglomerates that we observe in the present measurements are in the size range of agglutinates. Thus, one can conclude that, for SANS, fluorination does not change the size of individual DNDs and the size and structure of primary agglomerates.

### 3.3. SEM

**Figure 6:**
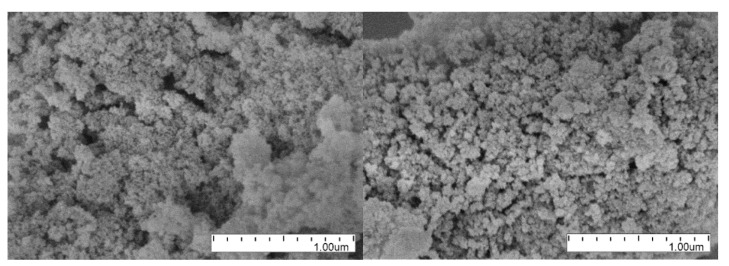
Examples of SEM images of DNDs (**on left**) and F-DNDs (**on right**).

Figure 6 illustrates the presence of clusters of DNDs in both raw DND and F-DND samples. We selected two out of many available images measured using the SEM. There are no visible modifications of the powder structure due to the fluorination process. The nanopowder structure features were examined by an ultra-high-resolution microscope Hitachi SU8020 equipped with a cold field cathode. DND samples were examined on the aluminum stage using a silver glue and coated with a thin layer of gold-palladium alloy by magnetron sputtering to make it electrically conductive. The layer thickness is ~20 nm, but it is not uniform. 

### 3.4. X-ray Powder Diffraction

Unlike SANS, XRD only measures diamond cores of DNDs. Amorphous shells of *sp*^2^ C atoms onto DND cores do not contribute to the diffraction signal. Thus, we performed such a measurement to verify the effect of fluorination on diamond cores and estimate the mean size of the cores.

XRD and SAXS data from powders in quartz capillaries were collected at the ID23 beamline at ESRF using a PILATUS 6 M area detector with 0.6888 Å wavelength X-rays. Data are reduced using the SNBL toolbox [57] and Fit2 D [58] software.

**Figure 7:**
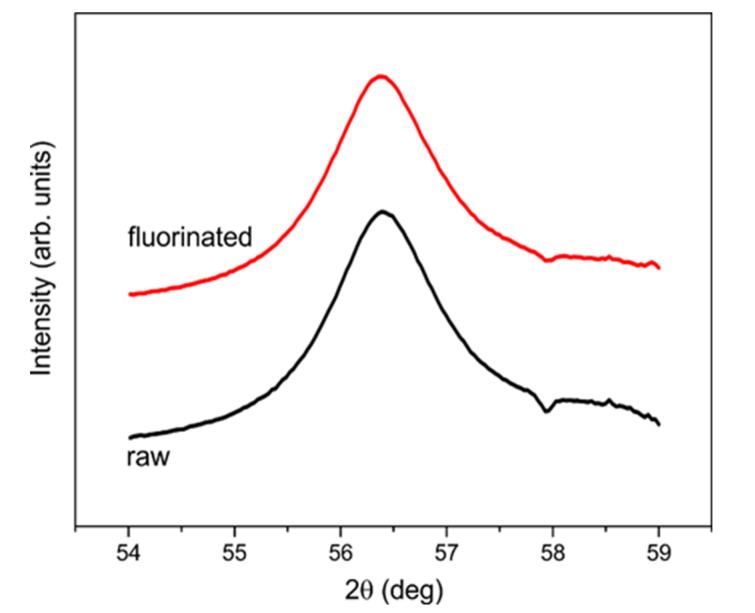
Powder diffraction for DND (black lower line) and F-DND (red upper line, shifted in intensity for better visibility) samples in the proximity of a 422 Debye-Scherrer ring. Full width at half maximum (FWHM) from the Lorentz line shape fit were evaluated to 1.343 ± 0.018 and 1.354 ± 0.022 for DND and F-DND samples, respectively, therefore coinciding. The mean size of DND cores is 4.3 nm.

The lattice spacing parameters nearly coincide with those for bulk diamond crystal; they are smaller by ~10^−3^. The four hundred and twenty-two powder diffraction line indicates the smallest spacing and allows the best sensitivity to the particle size effect to be achieved. The comparison of the lines for DNDs and F-DNDs shows that the coherent scattering regions, presumably coinciding with the DND cores, remain intact upon the fluorination (Figure 7).

## 4. Discussion

The virtual absence of a fluorination effect on scattering properties of DNDs was not obvious until the present experimental study and therefore it deserves a discussion. We know certain information about the changes of DND composition upon fluorination. The mass of DNDs remains almost unchanged, while H and amorphous *sp*^2^ shell of the DND were almost completely eliminated [27]. The ratio of F and C atoms in F-DND is F/C = 0.09 [29]. The fluorination at least partially removes one more major impurity, and namely oxygen, in which the fraction can reach 10% [59]. The fraction of N may decrease, but it has not been studied quantitatively. The neutron activation analysis showed that the composition and amount of metallic impurities in powders did not change noticeably [27].

Based on these data, we have to assume that the presence of F compensates the removal/reduction of *sp*^2^ C, H, O, N, and other elements. The coherent scattering cross section of the DND is proportional to the square of the sum of corresponding scattering lengths of all nuclei in the DND. The scattering lengths of these elements are 5.65 fm on one side and 6.65, −3.74, 5.80, and 9.36 fm on the other one, respectively. The fractions of elements required for such a compensation are compatible with the existing experimental data. However, this compensation is not sufficient. The angular characteristics of neutron scattering are determined by the shape and size of the scatterers in the DND. The XRD data show that diamond cores do not change upon the fluorination. The SANS data show that the fluorination does not change the shape and characteristic sizes of DNDs as well taking into account the impurity layer on their surface. 

On the other hand, the prompt-γ analysis revealed the large reduction of the probability of incoherent scattering due to the 30-fold reduction of the fraction of H, with its exceptionally large incoherent scattering cross section σ^H^_in.sc._ = 108 ± 2 b. This effect has led to a major reduction in neutron losses during neutron transport in powder, but to a much smaller effect on the results of SANS and a negligible effect on the results of XRD. The SANS data for DND and F-DND shown in Figure 3 are compatible with this statement. Incoherent scattering of neutrons by H explains the high scattering intensity I(Q) only at Q > 7·× 10^0^ nm. This difference of the scattering curves for DND and F-DND is compatible with the reduction of H upon fluorination observed in reference [27]. 

As it was noted in several publications [19,20,21], the agglomeration/clustering of DNDs plays a major role in scattering properties of DND powders. In particular, clustering increases straightforward scattering and at the same time it decreases the fraction of “visible” small DNDs, which define neutron scattering to large angles relevant to neutron reflectors. In terms of the model of discrete-size diamond nanospheres, clustering decreases the effective mass of powder. The rest mass contributes to losses but not to the scattering thus decreasing the efficiency of the reflector. In order to estimate the scale of this problem and investigate methods on how to tackle it, provided it is important, a dedicated study will be performed by us in the near future. 

## 5. Conclusions

We have studied the effect of the fluorination of DNDs on their structure and clustering, and found that it does not significantly affect both small-angle neutron scattering on individual DNDs as well as sizes and structure of clusters in the radii range of 0.6−200 nm. We conclude that the overall increase in neutron reflectivity is primarily due to the large reduction of neutron losses in the powder caused by the replacement of H by F.

To analyze the neutron scattering data, we developed a model of discrete-size diamond nanospheres. It is easy to use and can be applied in the future as a universal tool to compare results of measurements with other nanodiamond samples. 

We have confirmed that the effect of clustering of DNDs is significant when analyzing neutron scattering and neutron transport in DNDs powders. Further studies are required to understand how to reduce the clustering to improve the reflection efficiency of slow neutrons by DND powders.
